# A rare case of omental cake

**DOI:** 10.1259/bjrcr.20180042

**Published:** 2018-09-08

**Authors:** Natasha Gardiner, Peter Gonda, Christopher James, Haseeba Tawfeeq, Chukwumobi Ihezue

**Affiliations:** 1 Queen Alexandra Hospital, Portsmouth, England

## Abstract

Systemic amyloidosis is normally detected on histology by deposition of amyloid into soft tissue organs. Omental and peritoneal involvement are rare manifestations of the disease. The differential diagnosis for omental and peritoneal disease does not, therefore, normally include amyloid.

## Clinical presentation

A 59-yearold female patient was referred to the endoscopy department with a history of a small amount of fresh haematemesis during an episode of vomiting with a stomach bug. There was no weight loss or abdominal pain. The patient had a past medical history of myeloma with normal renal function and calcium. A hysterectomy had been carried out due to fibroids. The patient denied any history of asbestos exposure and any history of mesothelioma.

The initial endoscopy demonstrated lumpy gastritis in the cardia and fundus. Biopsies were taken and a repeat scope was arranged 4 weeks later to ensure healing. The histology from the biopsies revealed blue rubber bleb naevus disease. A second endoscopy was carried out to check if the bleeding had settled. It showed continued bleeding from the blebs. The patient was treated with an intravenous infusion of a proton pump inhibitor and remained stable.

## Differential diagnosis

1. Disseminated ovarian neoplasm
2. Abdominal mesothelioma
3. GI carcinomatosis
4. Peritoneal tuberculosis
5. Amyloidosis

## Investigations/Imaging findings

Following discussion in the upper gastrointestinal MDT, a volumetric CT chest abdomen pelvis was acquired, thereby allowing multiplanar reformats, to further investigate the blue blebs and whether there was an underlying lesion present. The chest and upper abdomen were imaged in the arterial phase and the entire abdomen and pelvis in the portal venous phase. Interestingly, the literature does not show any link of the blue rubber bleb naevus disease to malignancy but severe complications are gastrointestinal bleeding and soft tissue haematomas.^1^

**Figure 1.**
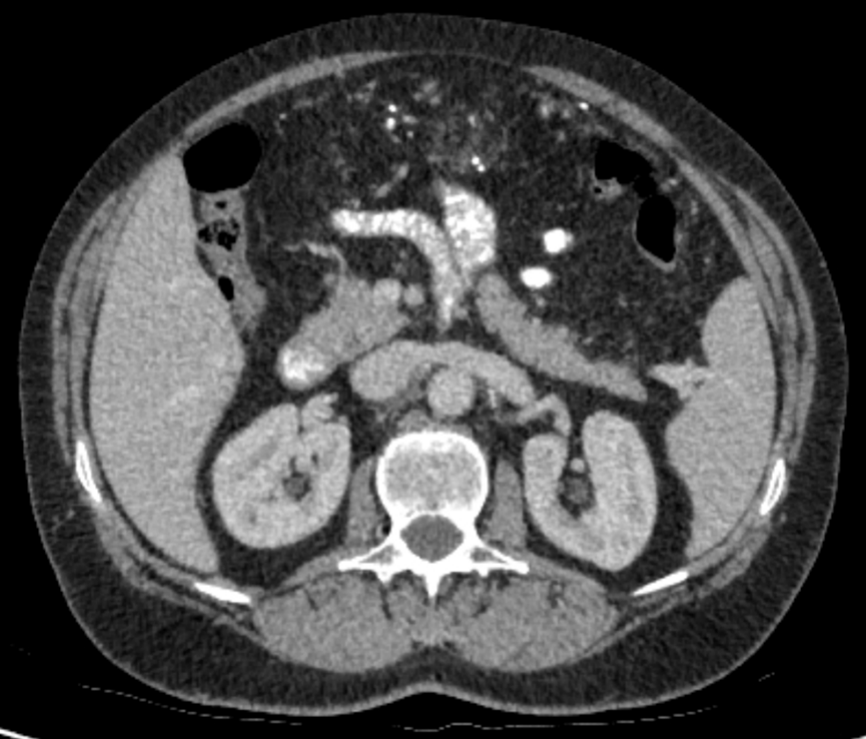
CT axial slice of upper/lower abdomen showing thickening of the omentum which contains a mantle of speckled calcifications.

**Figure 2.**
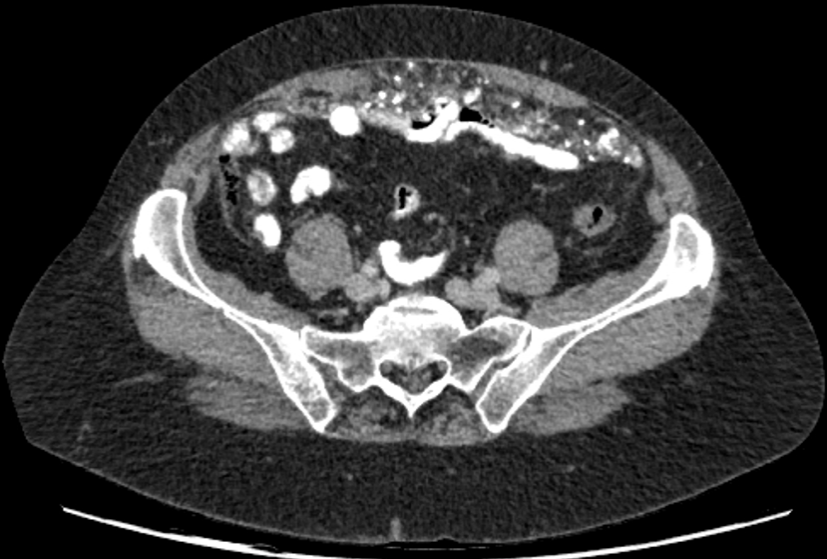
CT axial slice of upper pelvis showing thickening of the omentum which contains a mantle of speckled calcifications.

The CT showed no mass in the stomach, oesophagus, small bowel or large bowel, or any enlarged lymph nodes. There was evidence of a hysterectomy and bilateral salpingo-oophorectomy and no evidence of calcified asbestos exposure. However, it showed diffuse infiltration of the omental fat by material of soft tissue and calcified density. This is known as omental cake as it refers to a contiguous omental mass, which simulates the top of a cake (Figures 1 and 2).^2^

Following this, the patient was referred to the gynaecological team.

The Ca 125 was only marginally raised at 45 U ml^−1^ (normal <35). Therefore, a biopsy of the omental cake was organised to try and determine the histological nature of this omental mass.

## Treatment

**Figure 3.**
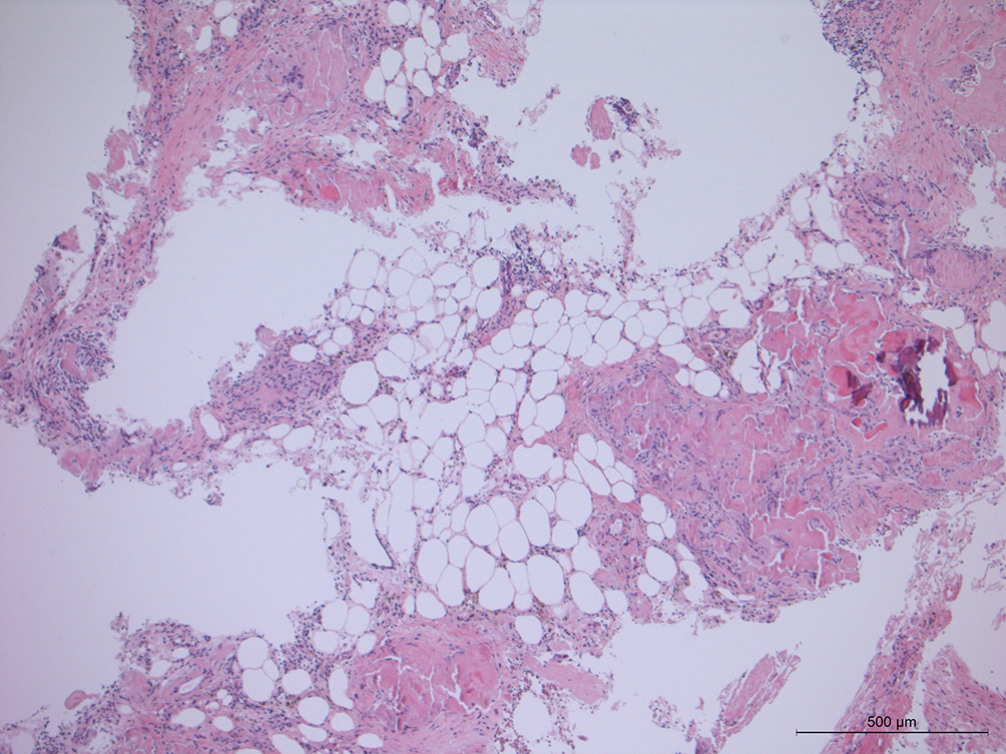
AL amyloid staining of a biopsy of the thickened omentum (HE 50×).

**Figure 4.**
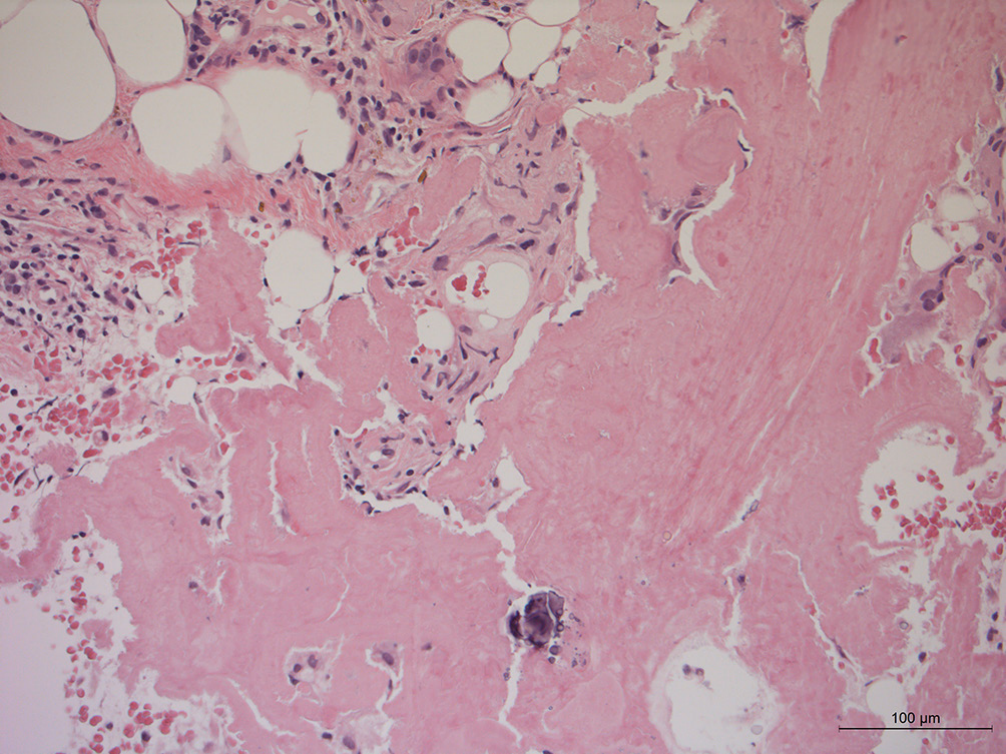
AL amyloid staining of a biopsy of the thickened omentum (HE 200×)

The omental biopsy demonstrated positive AL (light-chain) amyloid staining but no neoplastic cells (Figures 3 and 4).

**Figure 5.**
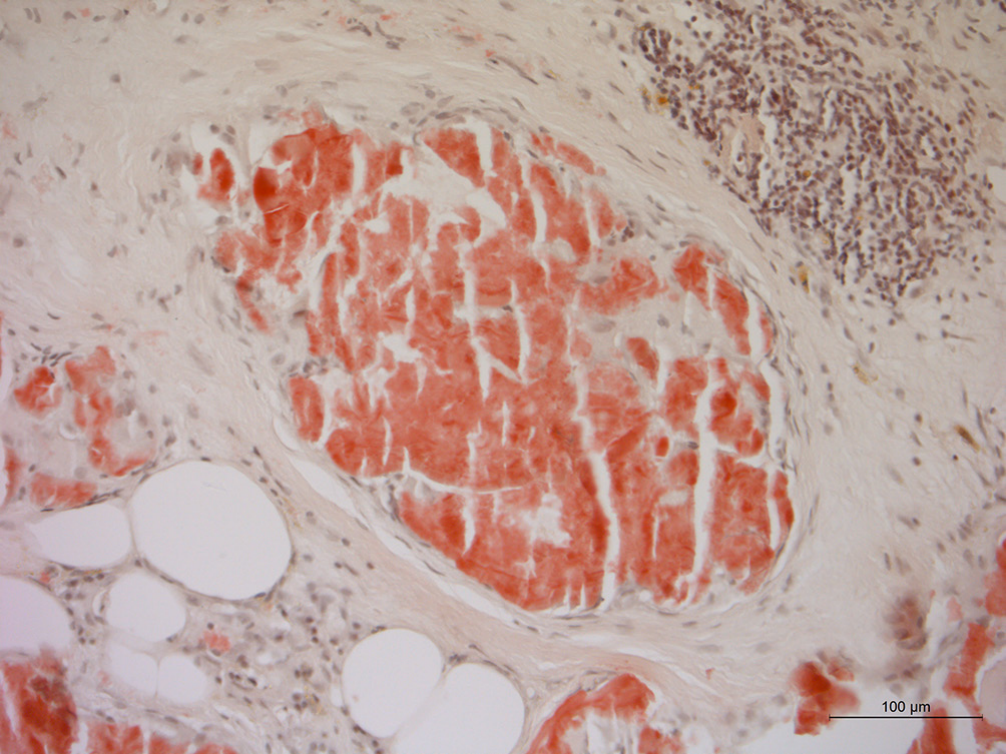
Congo red staining of a biopsy of the thickened omentum (200× non-polarised).

**Figure 6.**
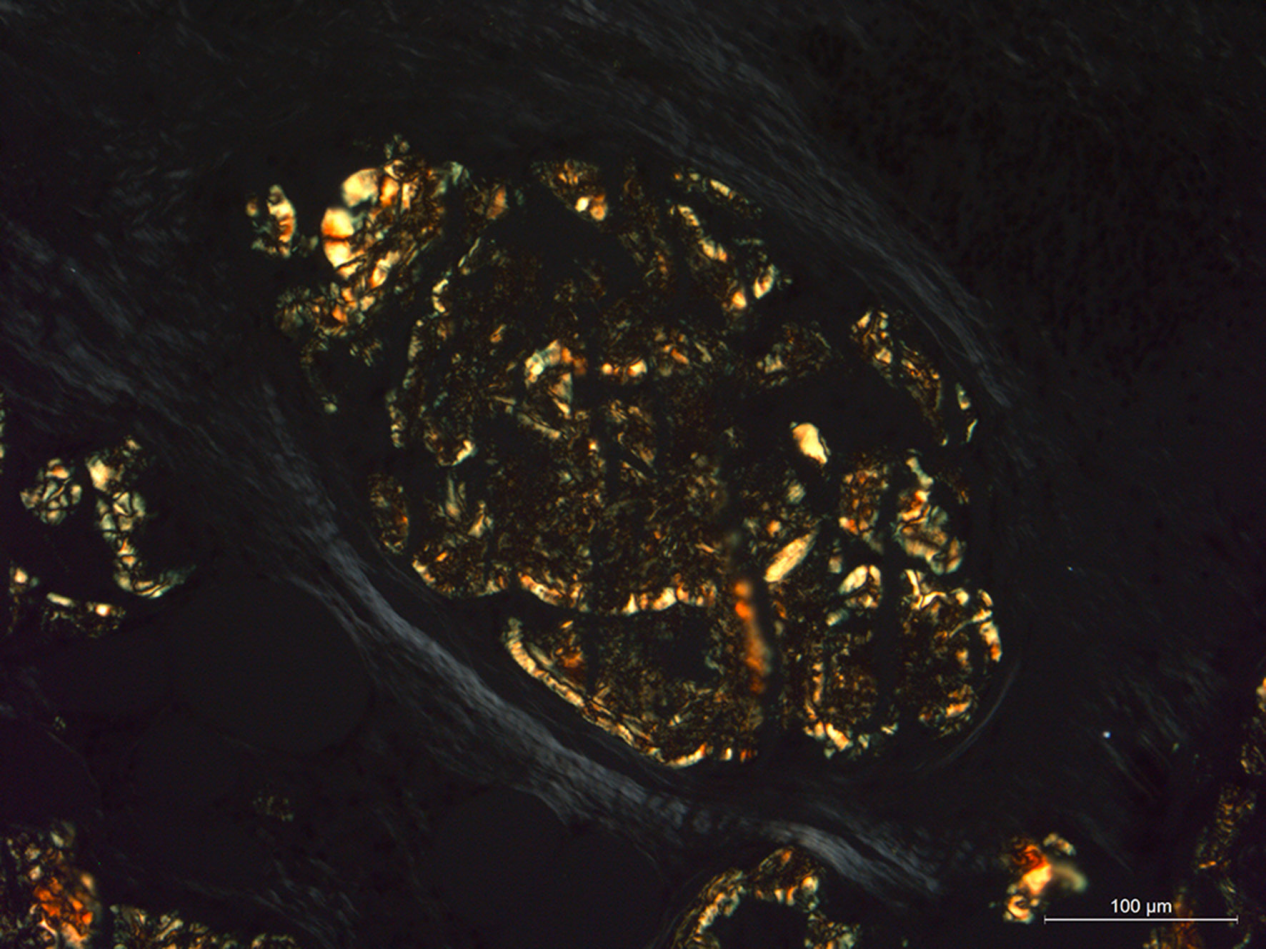
Congo red staining of a biopsy of the thickened omentum (200× polarised)

On Congo Red staining with polarized light the apple green appearance was found, supporting the amyloid diagnosis (Figures 5 and 6).

Following this, the patient was referred to the National Amyloidosis Centre. An echo was carried out which showed that there was no evidence of cardiac amyloidosis and SAP (Serum Amyloid P component) scintigraphy confirmed no visceral amyloid. The patient was treated with five cycles of velcade, cyclophosphamide and dexamethasone. In this case, the CT images of the calcified omental disease did not show improvement, despite the normalisation of the patient’s serum free light chains and IgG λ paraprotein.

## Discussion

Amyloidosis is a disorder of protein folding.^3^ The disease becomes significant when organ function is damaged due to the deposition of amyloid fibrils^4^ in multiple organs and tissues.^5^ In 2008, the annual incidence of amyloidosis in the UK was approximately 0.8/100000 of the population.^3^ Amylodosis is currently classified chemically. The specific amyloid protein present within the tissue is detected. There are five systemic forms of amyloid (four acquired and one hereditary)—AL, AA, Aß2M, and two ATTR types.

Firstly, and in this case, acquired AL amyloidosis is caused by plasma cell dyscrasias. The precursor protein of this type is a κ or λ immunoglobulin light chain. Clinically, this type can present involving different body systems, such as in cardiomyopathy, hepatomegaly and nephrotic syndrome. It has been shown that up to 20% of AL patients have multiple myeloma.^6^ Secondly, acquired AA is caused by chronic inflammation and is due to the precursor protein serum amyloid A. The most common clinical features of this type involve the renal system and are namely proteinuria and loss of renal function. Thirdly, acquired Aß2M amyloidosis is caused by long-term haemodialysis with high levels of the precursor protein ß2-microglobulin. Clinical characteristics are carpal tunnel syndrome and joint problems, involving the shoulders and wrists, for example. Fourthly, acquired ATTR amyloidosis is found in older people, commonly over the age of 80. The precursor protein is transthyretin and presents with a slowly progressive cardiomyopathy. Lastly, hereditary ATTR amyloidosis is a result of at least 80 autosomal dominant hereditary point mutations of the precursor protein transthyretin. Clinical characteristics of this type are peripheral and autonomic neuropathy, but also include cardiac, renal, and ocular involvement.^7^

Peritoneal calcification is rarely seen on CT of the abdomen and pelvis and is usually, as in our case, asymptomatic.^8^ When it is present, it has a number of both benign and malignant causes. Malignant causes are often accompanied by calcified lymph nodes, and include gynaecologic, squamous cell lung cancer and melanoma, which can predispose to paraneoplastic hyperparathyroidism and hypercalcaemia, colon cancer and gastric cancer. Benign causes demonstrate sheet like calcification and include AIDS, extraperitoneal pneumocystis infection and tuberculosis.^9^

CT findings of peritoneal amyloidosis are very similar to peritoneal carcinomatosis. There are two different patterns of presentation—nodular and diffuse. A nodular pattern depicts mesenteric masses and localized intestinal wall thickening. In our case, the diffuse pattern is demonstrated, and diffuse peritoneal thickening and irregular calcifications instead are displayed.^8^

The aim of amyloidosis treatment is to reduce the protein that results in the amyloid deposits. Treatment is more successful in AL amyloidosis.^8^ However, if there is multiorgan involvement the prognosis is poor.^10^

## Learning points

1. The differential diagnosis for CT appearances demonstrating calcified omental disease is wide and has both benign and malignant causes. Amyloidosis is a very rare cause.
2. Definitive diagnosis with a tissue sample is necessary.
3. Treatment is more successful in AL amyloid than the other types. Treatment response should be measured by monitoring the serum free light chains and IgG λ paraprotein. Radiological appearances should not be used as they may not, as in our case, change.
